# Outcomes of target therapies for pediatric BRAF V600-mutant low-grade gliomas: A systematic review and meta-analysis

**DOI:** 10.1093/noajnl/vdag192

**Published:** 2026-07-27

**Authors:** Katherine Figueira, Laura Elin Pigott

**Affiliations:** School of Allied Health and Life Sciences, College of Health and Life Sciences, London South Bank University, London, UK (K.F., L.E.P.); School of Allied Health and Life Sciences, College of Health and Life Sciences, London South Bank University, London, UK (K.F., L.E.P.)

**Keywords:** BRAF V600 mutation, functional outcomes, pediatric low-grade glioma, systematic review, targeted therapy

## Abstract

**Background:**

This systematic review and meta-analysis evaluated the efficacy, safety, and available functional outcomes of targeted therapies, including BRAF and MEK inhibitors, in pediatric patients with BRAF V600-mutant low-grade gliomas (pLGGs). The review also explored their impact on clinical management, recovery, and quality of life.

**Methods:**

The review followed PRISMA 2020 guidelines. Systematic searches were conducted across PubMed, ScienceDirect, EBSCO-hosted databases, and CENTRAL. Eligible studies included randomized controlled trials (RCTs) and retrospective studies (RS) reporting on tumor overall response rates (ORRs), progression-free survival (PFS), adverse events (AEs), and functional outcomes in patients aged 1-17 years. Risk of bias was assessed using CASP; the certainty of evidence was evaluated using the GRADE framework. Meta-analyses used a random-effects model (STATA 18.5). Funnel plots, Egger’s tests, and subgroup/sensitivity analyses explored bias and heterogeneity.

**Results:**

Ten studies (16 treatment arms; 4 RCTs, 6 RS were included). Pooled ORR was 42.35% (95% CI, 29.41-55.29), and the median PFS was 23.03 months (95% CI, 17.18-28.88). Grade 3/4 AEs occurred in 30.56% (95% CI, 22.60-38.53), most commonly rash, fatigue, and fever. Subgroup analyses suggested higher efficacy and lower toxicity with BRAF inhibitors or combination therapy. Functional outcomes were inconsistently reported, though some evidence suggested improvements in vision and performance status.

**Conclusions:**

Targeted therapies are effective and well tolerated in pediatric BRAF V600-mutant LGG, offering a promising alternative to chemotherapy, particularly in relapsed or progressive disease. Emerging evidence suggests potential benefit in selected molecularly defined patients; however, further prospective studies are required to define their role in first-line treatment. Standardized outcome reporting and long-term follow-up are essential to optimize care.

Key Points

- Targeted therapy is effective and well-tolerated in BRAF-mutant pediatric LGG.
- Molecular profiling may support earlier personalized care and reduce burden.
- Functional outcomes underreported, highlighting a key evidence gap.

Importance of the Study

This review highlights the potential of targeted therapies, particularly BRAF inhibitors, in the management of pediatric BRAF V600-mutant LGG. Findings suggest that molecularly guided treatment approaches may improve disease control while reducing treatment burden compared with conventional therapies. However, as most available evidence derives from relapsed or progressive disease, further prospective studies are needed to define the role of target therapies in earlier treatment settings and to inform future clinical guidelines.

**Background** 

Pediatric low-grade gliomas (pLGGs) are the most common brain tumors in children, accounting for 25%-30% of all pediatric brain and spinal cord tumors.^1^ Although pLGGs generally have favorable prognoses, with 10-year overall survival rates exceeding 90%,^2^ their management remains complex due to their chronic trajectory, recurrence, and treatment-related morbidity,^3^ particularly for tumors near critical structures, where surgical resection is often unfeasible.^1^

A significant advancement in the understanding of pLGGs has come from molecular diagnostics, particularly the identification of the BRAF V600 mutation, present in 15%-20% of cases.^3^^,^^4^ This mutation activates the RAS/RAF/MEK/ERK signaling pathway, promoting tumor growth.^4^^,^^5^ These tumors respond poorly to chemotherapy and carry a greater risk of malignant transformation compared to BRAF wild-type tumors.^2^^,^^6^ Up to 50% of these cases are refractory to conventional chemotherapy.^7^

Conventional treatments, including surgery, chemotherapy, and radiotherapy, carry significant adverse effects and suboptimal progression-free survival (PFS) rates.^8^^,^^9^ Long-term toxicities often affect quality of life and neurodevelopmental outcomes, especially in younger patients.^2^^,^^3^^,^^7^ This underscores the need for more effective and less toxic therapeutic options.

Targeted therapies, such as BRAF and MEK inhibitors, have emerged as promising alternatives by directly inhibiting the MAPK signaling pathway, the key driver of tumorigenesis in BRAF V600-mutant LGGs,^3^ which is commonly altered in Pilocytic astrocytoma, the most common pLGGs subtype.^1^^,^^10^ Clinical studies suggest that targeted therapies improve tumor response rates, extend PFS, and reduce treatment-related toxicities compared to chemotherapy.^2^^,^^11-13^

Assessing motor and cognitive functions is crucial, as pLGGs and their treatments often cause deficits in coordination, memory, and attention, affecting daily functioning and quality of life.^9^^,^^14^^,^^15^ Up to 50% of survivors face cognitive challenges that affect academic and social development,^7^^,^^16^ while motor deficits further impact daily functioning.^15^ These outcomes, often exacerbated by conventional therapies, underscore the importance of incorporating functional measures into treatment evaluation and research.^17^

Despite the growing number of studies on targeted therapies for gliomas, evidence specific to pediatric populations remains limited, fragmented, and extrapolated from adult populations.^3^ However, with the emergence of several new clinical trials and cohort studies in 2023 and 2024 focusing exclusively on children, there is now a timely need for an updated and focused synthesis. Existing reviews often prioritize tumor response rates, often underexploring broader clinical outcomes, including safety, quality of life, and functional impacts specific to pediatric patients.^7^ The rapidly evolving clinical landscape necessitates a pediatric-specific synthesis of evidence, marking the novel contribution of this systematic review.

Recent regulatory approvals by the US Food and Drug Administration and the National Institute for Health and Care Excellence (NICE) for the use of Dabrafenib and Trametinib in pediatric BRAF V600-mutant LGGs,^3^ alongside trials such as NCT02684058 and NCT05566795, reflect the growing relevance of targeted therapies. However, data on long-term and functional population remain limited. This systematic review and meta-analysis provide the first comprehensive synthesis of both clinical and functional outcomes in pediatric patients with BRAF V600-mutant LGGs.

## Methods

### Study Design

This systematic review and meta-analysis was conducted in accordance with the PRISMA 2020 guidelines^18^ and the Cochrane Handbook for Systematic Reviews of Interventions.^19^ The review protocol was developed using the PICO framework, outlining the research question, eligibility criteria, and outcome measures (Table S2).

The objective was to evaluate the efficacy, safety, and explore the available evidence on functional outcomes of targeted therapies in pediatric patients with BRAF V600-mutant low-grade glioma (LGG). The review included both randomized controlled trials (RCTs) and retrospective studies (RS), enabling the integration of controlled and real-world evidence.

### Search Strategy

A systematic search was conducted across PubMed, Science Direct, the Cochrane Central Register of Controlled Trials (CENTRAL), and EBSCO-hosted databases (including MEDLINE, CINAHL, and others) on December 20, 2024, without date restrictions (Supplementary Material S2). The search combined MeSH terms and keywords related to “BRAF,” “V600,” “paediatric,” “glioma,” “low-grade,” “dabrafenib,” “trametinib,” and “targeted therapy.” Boolean operators (AND/OR), truncation symbols, and filters for pediatric populations and publications from 2019 onward were applied. Manual reference screening of all included articles was also performed to identify any additional eligible studies.

### Eligibility Criteria

Studies were included if they met the following criteria: (1) enrolled patients aged 1-17 years with confirmed BRAF V600-mutant LGG; (2) investigated BRAF or MEK inhibitors, either as monotherapy or in combination; (3) reported on efficacy (eg overall response rate [ORR], PFS), safety (eg adverse events [AEs] of any grade), or functional outcomes (eg vision, quality of life); and (4) used clinical trial or observational study designs (RCTs, cohorts, or case series with ≥5 patients). Exclusion criteria included non-targeted therapy, non-BRAF V600 mutations, adult-only studies, conference abstracts without full-text, reviews, and non-English, Spanish, or Portuguese publications.

### Data Extraction

A standardized data extraction form was used to collect study characteristics (author, year, design, setting), participant details (age, sample size, gender), diagnosis, prior treatments, intervention type (drug class, dose, duration), comparators, and outcomes. Extracted outcomes included tumor response (CR, PR, SD), PFS, AEs (any grade and grade 3/4), and functional outcomes (eg vision, QoL, cognition, motor skills) where reported. Data were independently reviewed for accuracy and completeness prior to synthesis.

### Quality Appraisal and Certainty

Risk of bias was assessed using CASP tools appropriate to each study design (RCT or cohort),^20^^,^^21^ with 2 independent reviewers resolving disagreements by discussion. Overall certainty of evidence was evaluated using the GRADE framework across 5 domains: risk of bias, inconsistency, indirectness, imprecision, and publication bias.^22^ Studies rated as high risk or with incomplete data were flagged for sensitivity analysis. Funnel plots and Egger’s regression tests were conducted in STATA 18.5 to assess publication bias for key outcomes (ORR, PFS, and grade 3/4 AE). Asymmetry and significant small-study effects were considered when applying GRADE downgrades.

### Statistical Analysis

Meta-analyses were conducted using STATA 18.5 (StataCorp, College Station, TX, United States). Pooled estimates for ORR, PFS, and grade 3/4 AEs were calculated using a random-effects model to account for anticipated heterogeneity across study designs and interventions. When SEs or CIs were not reported, they were estimated using binomial proportion formulas or derived from available data ranges.

Heterogeneity was assessed using Cochran’s Q test and the *I*^2^ statistic, with *I*^2^ ≥50% and *P *< .10 indicating substantial heterogeneity. Subgroup analyses were performed by drug class (BRAF vs MEK vs combination), study design (RCT vs RS), and treatment type (monotherapy vs combination therapy) to explore sources of variation.

Sensitivity analyses were conducted to test the robustness of findings, including a leave-one-out method for studies where confidence intervals were estimated, and exclusion of high-risk-of-bias studies based on CASP assessment.

All statistical tests were 2-tailed, and a *P*-value <.05 was considered statistically significant.

## Results

### Study Selection

**Figure 1.**
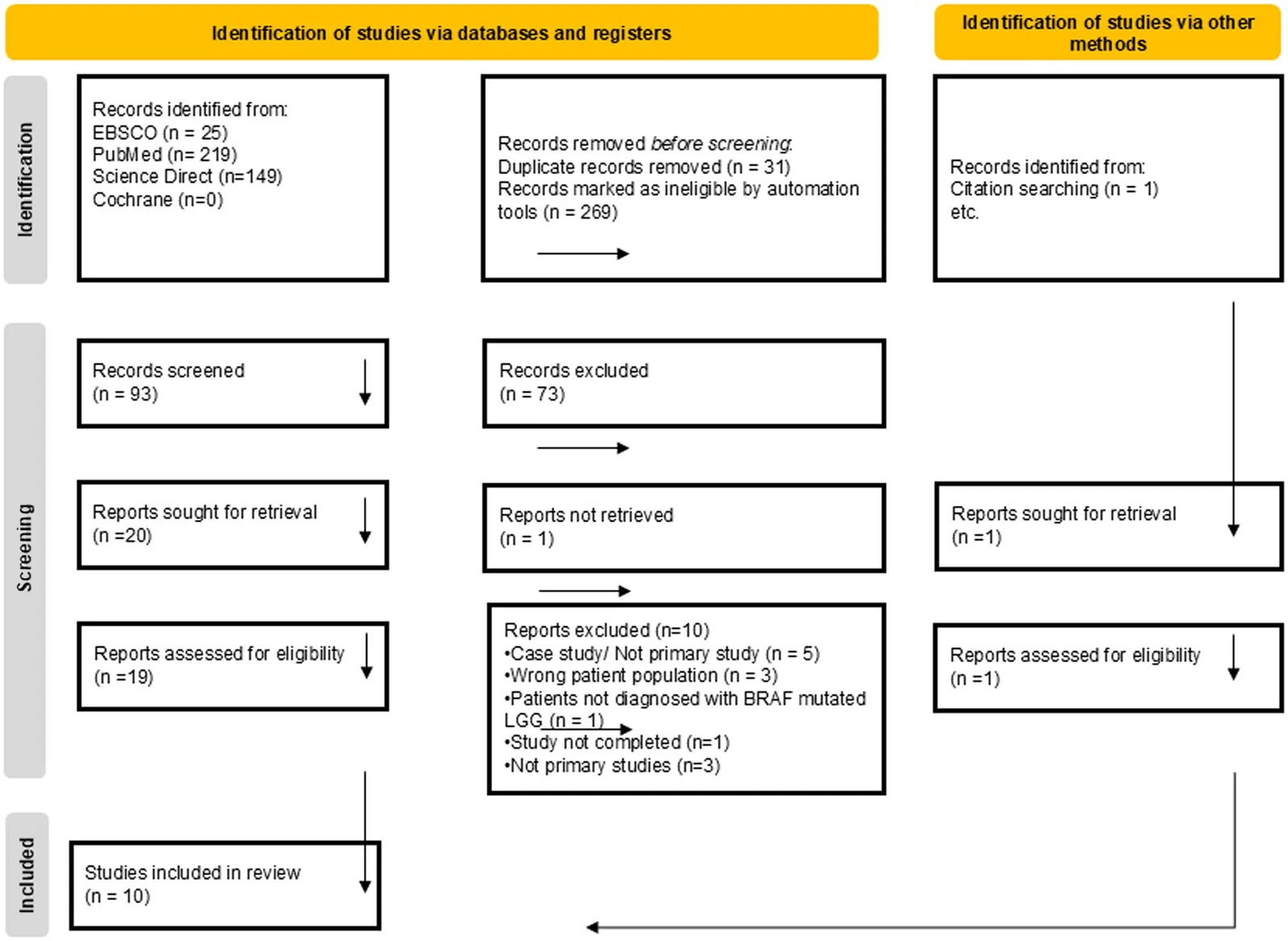
PRISMA 2020 flow diagram for new systematic reviews, which included searches of databases, registers, and other sources.

A total of 394 records were identified through database searches and citation tracking. After removing duplicates and ineligible records, 93 studies remained for title and abstract screening. Of these, 20 full-text articles were assessed for eligibility. Following the exclusion of studies not meeting the criteria, 10 studies were included in the systematic review. The selection process is summarized in Figure 1.

### Study Characteristics

The 10 studies included in this systematic review comprised 4 RCTs and 6 RS, published between 2019 and 2023. Across all studies, the total number of patients receiving targeted therapy ranged from 1 to 137, while the subset of patients with confirmed BRAF V600-mutant LGG ranged from 4 to 77 per study.

Targeted therapies were predominantly administrated in the relapsed, refractory, or progressive disease setting, particularly in RS, where most patients had received prior treatments, including surgery, chemotherapy, and/or radiotherapy. However, 2 studies evaluated targeted therapy as a first-line intervention.^6^^,^^23^

For meta-analysis purposes, studies were organized into 16 treatment arms, based on therapy type (monotherapy or combination therapy) and drug class (BRAF inhibitor, MEK inhibitor, combination, or other), enabling a clear evaluation of efficacy (eg ORR, PFS), safety (eg grade 3/4 AEs), and functional outcomes. It also supported subgroup analyses by mechanism of action. A summary of study characteristics, intervention, outcomes, and quality appraisal is presented in Table 1.

**Table 1.** Study characteristics and quality appraisal

Study	Study design	Sample size BRAF V600-mutant pLGG	Age, mean (range)	Targeted therapy (drug class; therapy type)	First-line intervention	Outcomes	Quality appraisal (**CASP)**
Kilburn et al. 2024	RCT	77	9 (1-24)	Tovorafenib (BRAF; Monotherapy)	No	ORR, PFS, AEs, FO	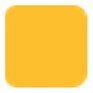
Bouffet et al. 2023	RCT	70	10 (1-17)	Dabrafenib + Trametinib (BRAF + MEK; Combination)	Yes	ORR, OS, PFS, AEs, FO	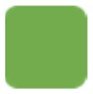
Bouffet et al. 2023	RCT	47	Monotherapy: 7 (2-17) Combination: 10 (1.4-17)	Trametinib (MEK inhibitor; Monotherapy)	No	ORR, PFS, AEs	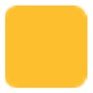
				Dabrafenib + Trametinib (BRAF + MEK inhibitor; Combination)			
Hargrave et al. 2019	RCT	32	8.5 (2-17)	Dabrafenib (BRAF; Monotherapy)	No	ORR, PFS, AEs, FO	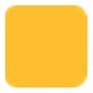
Selt et al. 2020	RS	10	8.2 (3.5-17.3)	Trametinib (MEK; Monotherapy)	No	ORR, AEs	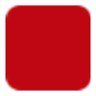
Leclair et al. 2022	RS	5	4.5 (3.1-9.5)	Dabrafenib (BRAF; Monotherapy)	Yes	ORR, AEs	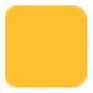
				Trametinib (MEK; Monotherapy)			
Perez et al. 2021	RS	23	Trametinib: 1.6 (0.4-5.9) Dabrafenib: 6.2 (0.5-17.8)	Trametinib (MEK; Monotherapy)	No	ORR, PFS, AEs, FO	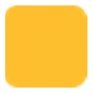
				Dabrafenib (BRAF; Monotherapy)			
Nobre et al. 2020	RS	56	8.2 (NA)	Dabrafenib (BRAF; Monotherapy)	No (mostly)	ORR, PFS, AEs, FO	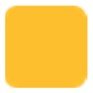
				Vemurafenib (BRAF; Monotherapy)			
Tsai et al. 2023	RS	55	4.42 (0.13-25.84)	Dabrafenib (BRAF; Monotherapy)	No	ORR, PFS, AEs, FO	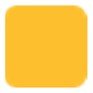
				Vemurafenib (BRAF; Monotherapy)			
				Trametinib (MEK; Monotherapy)			
				Everolimus (Other; Monotherapy)			
Manoharan et al. 2020	RS	11	14.7 (7.3-25.9)	Trametinib (MEK; Monotherapy)	No	ORR, AEs	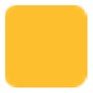

Abbreviations: AE, adverse event; BRAF V600E, B-rapidly accelerated fibrosarcoma V600 mutation; FO, functional outcome; MEKi, MEK inhibitor; NA, not available; ORR, objective response rate; PFS, progression-free survival; pLGG, pediatric low-grade glioma; RCT, randomized controlled trial; RS, retrospective study. Red box, high risk of bias;  Yellow boxes, moderate risk of bias;  Green box, low risk of bias.

### Quality Appraisal

Among the 10 included studies, CASP ratings indicated low risk of bias for all 4 RCTs and moderate to high risk for 6 RS, primarily due to lack of randomization, blinding, and incomplete outcome data. Functional outcomes were inconsistently reported, limiting interpretability. GRADE assessments rated the certainty of evidence for ORR and PFS as high in RCTs but very low to moderate in cohort studies due to concerns about inconsistency, indirectness, and imprecision. For grade 3/4 AEs, certainty was moderate for RCTs and very low for RSs. Functional outcomes could not be formally graded due to insufficient data. The traffic light quality appraisal is presented in Table 1, with a detailed breakdown of CASP and GRADE in Table S4. Funnel plots and Egger’s tests assessed publication bias for ORR, PFS, and grade 3/4 AEs. For ORR, Egger’s test showed no significant small-study effects (*P* = .727), though some visual asymmetry was noted. PFS showed no significant bias (*P* = .071), but the funnel plot suggested mild asymmetry, possibly reflecting heterogeneity in study design and reporting. For grade 3/4 AEs, Egger’s test approached significance (*P* = .109), and the funnel plot showed moderate asymmetry, suggesting possible small-study or reporting bias.

**Figure 2.**
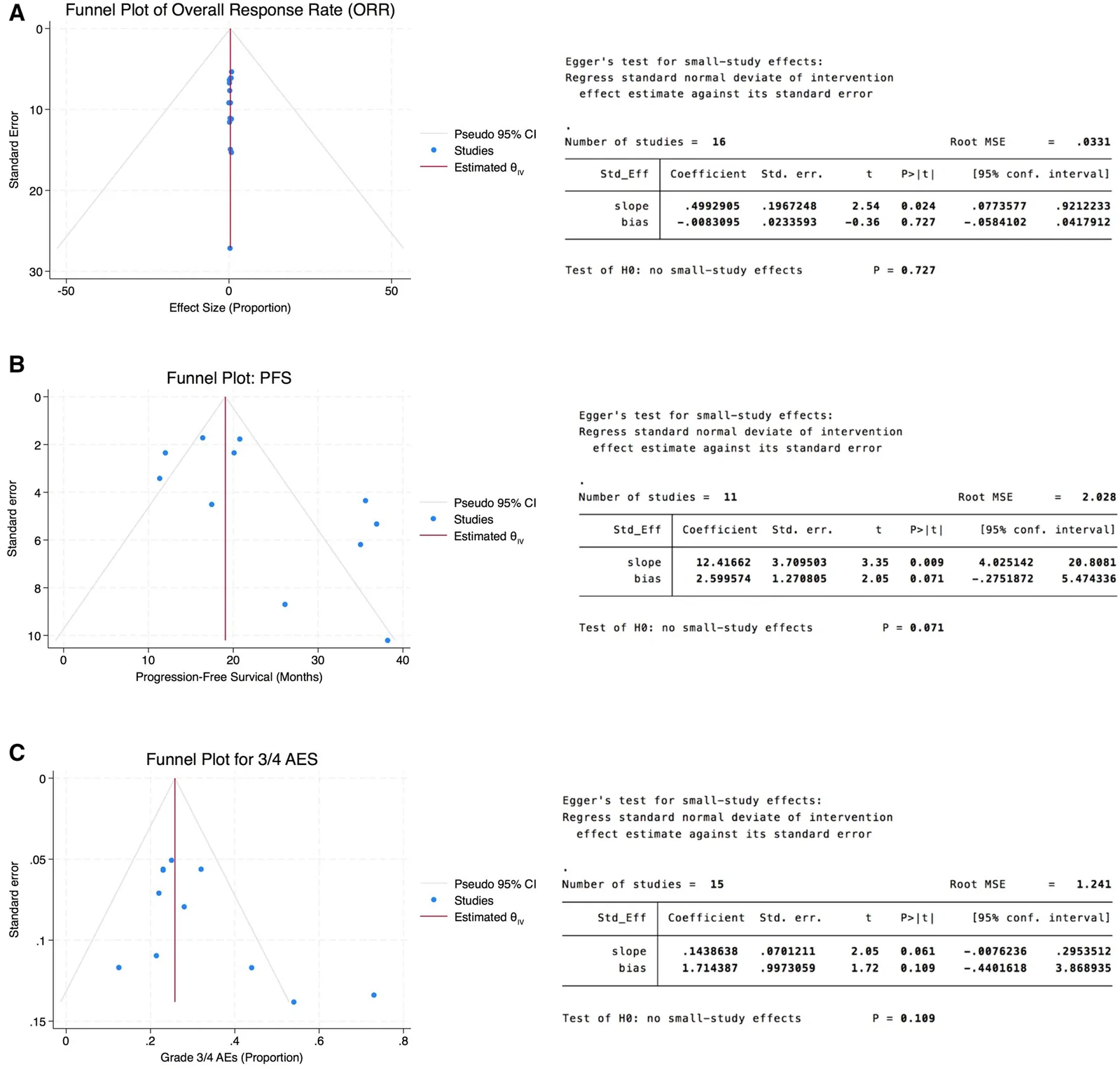
Funnel plots and Egger’s test for publication bias. (A) Funnel plot for overall response rate (ORR), showing treatment arms plotted by effect size (proportion) and standard error. Egger’s test (right) showed no significant small-study effects (*P* = .727). (B) Funnel plot for progression-free survival (PFS), with effect sizes expressed in months. Egger’s test showed marginal asymmetry (*P* = .071), suggesting a potential small-study effect. (C) Funnel plot for grade 3/4 adverse events (AEs), using proportion data. Egger’s test indicated no significant publication bias (*P* = .109). Each blue dot represents a treatment arm. The red vertical line indicates the estimated pooled effect. Funnel symmetry supports the absence of publication bias, whereas asymmetry may indicate selective reporting.

While statistical tests did not confirm publication bias, visual inspection raises caution, particularly for safety outcomes. Funnel plots and test results are presented in Figure 2.

## Meta-Analysis Findings

### Outcome Efficacy

#### Overall Response Rate

**Figure 3.**
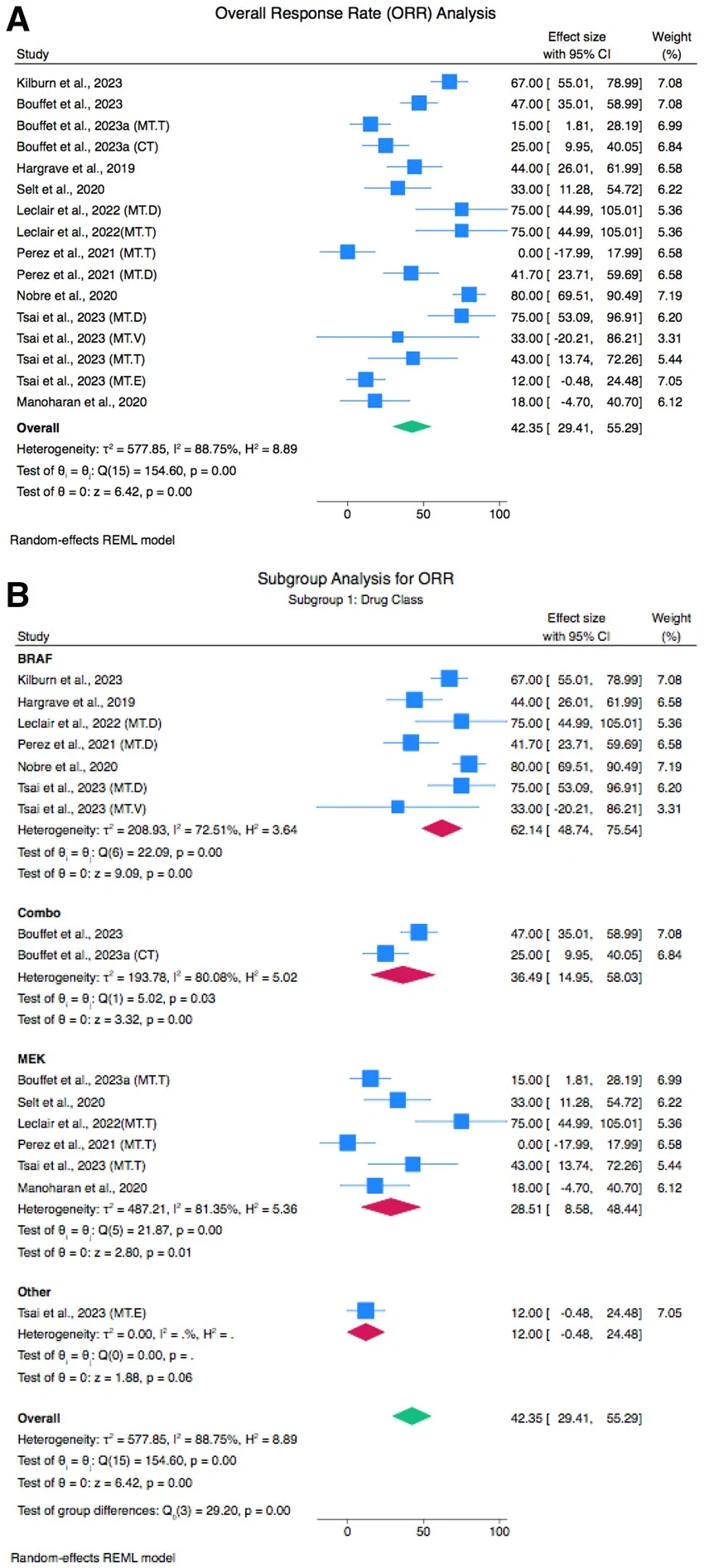
Overall response rate (ORR) in pediatric BRAF V600-mutant LGG. (A) Forest plot of ORR across 16 treatments arms using a random-effects model. (B) Subgroup analysis of ORR by drug class (BRAF, Combination, MEK, other).

The pooled ORR was 42.35% (95% CI, 29.41%-55.29%; *P* < .001), with substantial heterogeneity (*I *^2^ = 88.75%) (Figure 3A). Subgroup analysis revealed that BRAF inhibitor monotherapy showed the highest pooled ORR (62.14%), followed by combination therapy (36.49%) and MEK inhibitor monotherapy (28.51%) (Figure 3B). There was no statistically significant difference in ORR between studies based on study design (RCT vs RS) or therapy type (monotherapy vs combination therapy) (Figure S1).

#### Progression-Free Survival

**Figure 4.**
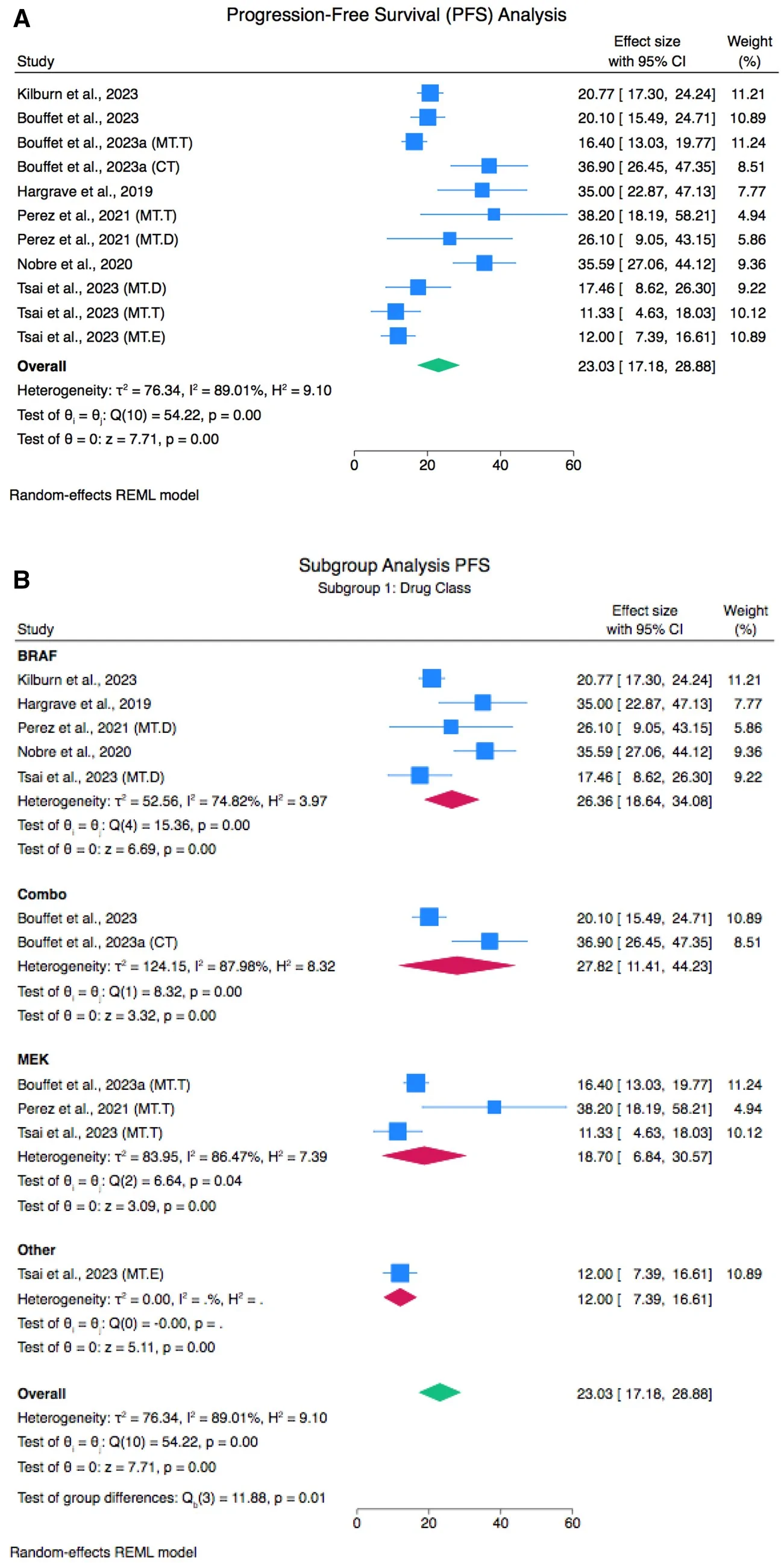
Progression-free survival (PFS) in pediatric BRAF V600-mutant LGG. (A) Forest plot of pooled median PFS across 11 treatment arms, calculated using a random-effects model. (B) Subgroup analysis of PFS by drug class (BRAF, Combination, MEK, other).

The pooled median PFS was 23.03 months (95% CI, 17.18-28.88), with high heterogeneity (*I *^2^ = 89.01%) (Figure 4A). Combination therapy demonstrated the longest PFS (27.82 months), followed by BRAF inhibitor monotherapy (26.36 months) and MEK inhibitors (18.70 months) (Figure 4B). Subgroup analysis by drug class was statistically significant (*P *= .01), while differences by study design and therapy type were not (Figure S2).

### Safety Outcomes

**Figure 5.**
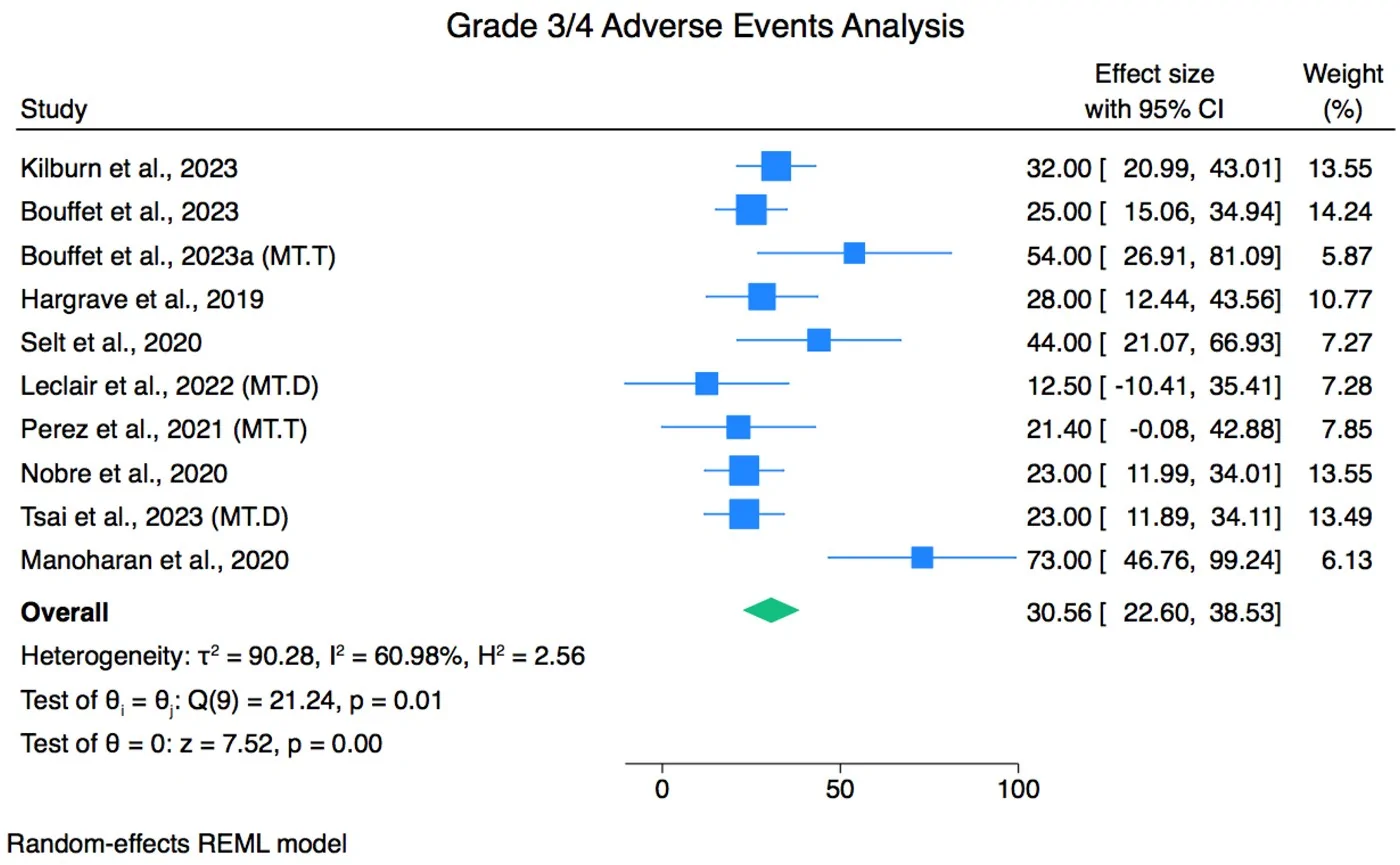
Forest plot of grade 3/4 Adverse Side Effects across 10 treatments arms using a random-effects model.

Any grade AEs ranged from 89% to 100%. The pooled incidence of grade 3/4 AEs was 30.56% (95% CI, 22.60%-38.53%; *P *< .001), with moderate heterogeneity (*I*^2^ = 60.98%) (Figure 5). Frequently reported toxicities included rash, fatigue, fever, and elevated creatine phosphokinase (CPK) levels. No statistically significant differences were observed in grade 3/4 AE rates by therapy type or study designs (Figure S3).

### Functional Outcomes

Functional outcomes were inconsistently reported and were therefore evaluated descriptively, with limited use of standardized tools. Vision-related outcomes were the most frequently documented, reported in 6 of 10 studies, with improvements observed in 4 (eg Bouffet et al. 2023; Perez et al. 2021). Bouffet et al. (2023) noted visual improvement in 34% of patients receiving targeted therapies, compared to 11% in the chemotherapy group. In contrast, Manoharan et al. (2020) reported no improvement in patients with preexisting optic nerve atrophy. Motor function was briefly mentioned in 2 studies, including observations of clinical improvement^24^ and reduced growth velocity.^25^ Quality of life was referenced narratively in 4 studies, though none used validated tools. Cognitive outcomes were not reported. Due to the heterogeneity in outcome measures and absence of standardized instruments (eg PedsQL, neurocognitive assessments), quantitative synthesis was not feasible. A summary of reported functional outcomes is provided in Table 2.

**Table 2.** Functional outcomes summary

Study	Design	Vision	Cognition	Motor	QoL	Outcome notes
Kilburn et al. 2023	RCT	Yes	No	Yes	Yes	Growth preserved; no decline in performance scores
Bouffet et al. 2023	RCT	Yes	No	No	Yes	34% visual improvement vs 11% in chemo group
Bouffet et al. 2023	RCT	No	No	No	No	Not reported
Hargrave et al. 2019	RCT	No	No	No	No	Not reported
Selt et al. 2020	RS	No	No	No	No	Not reported
Leclair et al. 2022	RS	Yes	No	No	No	2/3 patients improved; 1 stable vision
Perez et al. 2021	RS	Yes	No	Yes	Yes	2 patients had improved vision
Nobre et al. 2020	RS	Yes	No	No	Yes	Narrative mention of clinical improvement incl. vision
Tsai et al. 2023	RS	Yes	No	No	Yes	Some vision/seizure improvement mentioned
Manoharan et al. 2020	RS	Yes	No	No	No	No visual improvement in optic pathway glioma

Abbreviations: RCT, randomized controlled trials; RS, retrospective studies; QoL, quality of life.

### Sensitivity and Heterogeneity Analyses

Excluding studies at high risk of bias or with imputed CIs produced minimal impact on pooled estimates, suggesting robust findings (Figure S4). However, substantial heterogeneity persisted across outcomes. For ORR and PFS (*I*^ 2^ = 88.75% and 89.01%, respectively), variation likely stemmed from differences in therapy type, dosing, treatment setting, study design, and age range (3.2-14.7 years). Therefore, pooled estimates should be interpreted cautiously given the substantial clinical and statistical heterogeneity across included studies.

## Discussion

This systematic review and meta-analysis evaluated the efficacy and safety of targeted therapies in pediatric patients with BRAF V600-mutant LGGs. The pooled analysis revealed an ORR of 42.35%, a median PFS of 23.03 months, and an occurrence of AEs of any grade reported across all studies, with a grade 3/4 AEs rate of 30.56%, with statistically significant results (*P *< .05). These findings suggest that targeted therapies offer a promising therapeutic avenue for this patient population, demonstrating moderate efficacy with a manageable safety profile.

### Efficacy Outcomes

The ORR of 42.35% indicates that nearly half of the treated patient experienced a significant reduction in tumor size, notably exceeding rates reported with chemotherapy.^26-28^ For example, Bouffet et al. (2023)^29^ reported a response rate of 47% for Dabrafenib plus Trametinib, compared to only 11% with standard chemotherapy (carboplatin and vincristine), further supporting the superior efficacy of targeted agents in this context.

The median PFS of 23.03 months also exceeded typical outcomes of conventional chemotherapy, which range from 10 to 18 months in similar pediatric populations.^28^ Subgroup analysis identified a significant association between drug class and PFS (*P *= .01), with combination BRAF/MEK inhibition yielding the longest PFS (27.82 months), followed by BRAF inhibitor monotherapy (26.36 months). These findings align with existing evidence that combining targeted agents may enhance disease control and reduce resistance.^30^

### Safety Outcomes

Targeted therapies were generally well tolerated in pediatric patients, with the incidence of any-grade AEs ranging from 89% to 100% and grade 3/4 AEs of 30.56% (95% CI, 22.60%-38.53%). Common toxicities included dermatological toxicities (eg rash, dry skin, maculopapular rash), fatigue, diarrhea, and elevated CPK levels.^31^ While differences in toxicity profiles between BRAF and MEK inhibitors have been reported in the literature,^32^ subgroup analyses in this review did not yield statistically significant results to support definitive conclusions regarding agent-specific AE patterns.

To manage treatment-related toxicities, Cacciotti et al. (2025) recommend proactive monitoring, including regular dermatological, ophthalmologic, cardiac, and hepatic assessments. These strategies are especially important in children to detect complications early, support adherence, and preserve long-term safety.

### Functional Outcome

Functional outcomes were inconsistently reported and could not be pooled for meta-analysis. Visual improvement was the most frequently assessed domain, reported in up to 34% of patients receiving targeted therapies, compared to 11% in chemotherapy-treated groups (Nobre et al. 2020). Some studies noted stability or improvement in performance status^29^; however, standardized tools such as the PedsQL or Karnofsky/Lansky scores were rarely used.^11^^,^^33^ Motor function, neurocognitive performance, and quality of life were largely absent or only qualitatively described, limiting comparability across studies.

Although clinicians have gained experience in managing and monitoring the toxicities of targeted therapies, key questions remain regarding long-term effects on neurodevelopment, growth, and psychological outcomes.^16^^,^^30^^,^^31^^,^^34^^,^^35^

While early findings suggest a potential functional benefit, particularly in preserving vision,^23^^,^^24^^,^^27^^,^^29^^,^^33^ the overall lack of robust data highlights a significant evidence gap. Future trials should incorporate standardized, validated measures to comprehensively assess the impact of targeted therapies on pediatric patients’ daily functioning and well-being.

### Clinical Implications and Impact on Guidelines

This review highlights that BRAF V600E-mutant pLGG is a distinct subtype with poorer prognosis and higher risk of malignant transformation, carrying important clinical and policy implications.^26^^,^^36^ Targeted therapies demonstrated improved tumor control with a generally manageable safety profile, particularly in relapsed or progressive cases.

Emerging evidence suggests potential benefits of BRAF inhibitors as a first-line treatment option^6^,^23^; however, most of the evidence included in this study evaluated this treatment after progression or relapse. Therefore, while early use appears promising, further studies are required before routine first-line implementation can be recommended.

Despite their promise, these therapies are primarily disease-controlling rather than curative, requiring prolonged use and carrying the risk of resistance over time.^35^^,^^37^ Effectiveness of BRAF and MEK inhibitors varies considerably across molecular subgroups and long-term outcomes remain uncertain.^33^^,^^38^ Further research is essential to better understand mechanisms of resistance and to optimize treatment sequencing and combination strategies.^35^^,^^37^

Recent updates to the NICE guidelines have acknowledged the role of targeted therapies, particularly Dabrafenib plus Trametinib in relapsed or refractory pLGG.^39^ The findings of this review suggest the potential benefits of molecularly guided treatment approaches and highlight the need for prospective studies evaluating targeted therapies in earlier treatment settings. Routine molecular profiling at diagnosis may help identify patients who could benefit from target therapies and inform treatment decision-making.^4^^,^^6^^,^^23^^,^^25^^,^^27^^,^^30^^,^^40^

Furthermore, future studies should routinely capture functional outcomes, including vision, cognitive function, and health-related quality of life, to better evaluate the long-term impact of these therapies on pediatric patients.^33^

### Strength and Limitations

This review is among the first to synthesize evidence on targeted therapies for pediatric BRAF V600-mutant LGG, using rigorous methods. Inclusion of randomized and RS, with subgroup and sensitivity analyses, strengthened breadth and interpretability. Limitations include reliance on small, retrospective, and heterogeneous studies, inconsistent functional outcome reporting, incomplete data, and high risk of bias from non-randomized designs. Variations in drug class, treatment setting, and patient characteristics contributed to substantial heterogeneity, and pooled estimates should therefore be interpreted cautiously. Nevertheless, these findings offer valuable insights and underscore the need for high-quality prospective studies with standardized outcomes.

## Conclusion and Recommendations

Targeted therapies, particularly BRAF inhibitors, appear effective and generally well-tolerated in children with BRAF V600-mutant LGGs, offering promising efficacy and fewer high-grade toxicities compared to chemotherapy. These highlight the potential role of molecularly stratified treatment pathways in clinical practice, although further prospective studies are needed to define the role in earlier treatment settings. Future research should focus on long-term, prospective studies that incorporate functional and patient-reported outcomes. Standardizing response criteria, AE grading, and follow-up duration will enhance comparability across studies. Further investigations are also needed to determine optimal duration, sequencing, and combination strategies to enhance long-term disease control and mitigate resistance.

## Supplementary Material

vdag192_Supplementary_Data

## Data Availability

The data supporting the findings of this study are available from the corresponding author upon reasonable request. As this study is based on data extracted from previously published studies, all primary source data are available within the cited articles. The data extraction forms and analyses generated during the current study are available from the corresponding author on reasonable request.

## References

[1] Al-Jilaihawi S , Lowis S. A molecular update and review of current trials in paediatric low-grade gliomas. Pediatr Neurosurg. 2023;58:290-298. 10.1159/000533703 37604126

[2] Plant-Fox AS , O’Halloran K, Goldman S. Pediatric brain tumors: the era of molecular diagnostics, targeted and immune-based therapeutics, and a focus on long term neurologic sequelae. Curr Probl Cancer. 2021;45:100777. 10.1016/j.currproblcancer.2021.100777 34303558

[3] Lei J , Liu Y, Fan Y. The effects of dabrafenib and/or trametinib treatment in Braf V600-mutant glioma: a systematic review and meta-analysis. Neurosurg Rev. 2024;47:458. 10.1007/s10143-024-02664-x 39172230 PMC11341626

[4] Ryall S , Zapotocky M, Fukuoka K, et al Integrated molecular and clinical analysis of 1,000 pediatric low-grade gliomas. Cancer Cell. 2020;37:569-583.e5. 10.1016/j.ccell.2020.03.011 32289278 PMC7169997

[5] Tateishi K , Nakamura T, Yamamoto T. Molecular genetics and therapeutic targets of pediatric low-grade gliomas. Brain Tumor Pathol. 2019;36:74-83. 10.1007/s10014-019-00340-3 30929113

[6] Bouffet E , Hansford JR, Garrè ML, et al Dabrafenib plus trametinib in pediatric glioma with BRAF V600 mutations. N Engl J Med. 2023;389:1108-1120. 10.1056/NEJMoa2303815 37733309

[7] Perreault S , Larouche V, Tabori U, et al A phase 2 study of trametinib for patients with pediatric glioma or plexiform neurofibroma with refractory tumor and activation of the MAPK/ERK pathway: TRAM-01. BMC Cancer. 2019;19:1250. 10.1186/s12885-019-6442-2 31881853 PMC6935133

[8] Bandopadhayay P , Bergthold G, London WB, et al Long-term outcome of 4,040 children diagnosed with pediatric low-grade gliomas: an analysis of the Surveillance Epidemiology and End Results (SEER) database. Pediatr Blood Cancer. 2014;61:1173-1179. 10.1002/pbc.24958 24482038 PMC4657506

[9] Fangusaro J , Jones DT, Packer RJ, et al Pediatric low-grade glioma: state-of-the-art and ongoing challenges. Neuro Oncol. 2024;26:25-37. 10.1093/neuonc/noad195 37944912 PMC10768984

[10] Hargrave DR , Bouffet E, Tabori U et al Efficacy and safety of dabrafenib in pediatric patients with BRAF V600 mutation-positive low-grade glioma: results from a phase I/IIa study. Clin Cancer Res. 2019;25:7303-7311. 10.1158/1078-0432.CCR-19-2177 31811016

[11] Manoharan N , Choi J, Chordas C, et al Trametinib for the treatment of recurrent/progressive pediatric low-grade glioma. J Neurooncol. 2020;149:253-262. 10.1007/s11060-020-03592-8 32780261

[12] Bouffet E , Hansford J, Garré ML, et al Primary analysis of a phase II trial of dabrafenib plus trametinib (dab + tram) in *BRAF* V600–mutant pediatric low-grade glioma (pLGG). JCO. 2022;40:LBA2002. 10.1200/JCO.2022.40.17_suppl.LBA2002

[13] Novartis Pharmaceuticals. Study of efficacy and safety of dabrafenib in combination with trametinib in pediatric patients with BRAF V600 mutation positive LGG or relapsed or refractory HGG tumors. ClinicalTrials.gov identifier Web site. https://clinicaltrials.gov/study/NCT02684058. Updated 2023. Accessed November 21, 2024.

[14] Heitzer AM , Ashford JM, Hastings C, et al Neuropsychological outcomes of patients with low-grade glioma diagnosed during the first year of life. J Neurooncol. 2019;141:413-420. 10.1007/s11060-018-03048-0 30467811 PMC6344293

[15] de Bont JM , Schouten-van Meeteren AY. Long-term quality of survival after pediatric low-grade glioma. Childs Nerv Syst. 2024;40:3341-3355. 10.1007/s00381-024-06631-1 39400717

[16] Armstrong GT , Liu Q, Yasui Y, et al Long-term outcomes among adult survivors of childhood central nervous system malignancies in the childhood cancer survivor study. J Natl Cancer Inst. 2009;101:946-958. 10.1093/jnci/djp148 19535780 PMC2704230

[17] Armstrong GT , Conklin HM, Huang S, et al Survival and long-term health and cognitive outcomes after low-grade glioma. Neuro Oncol. 2011;13:223-234. 10.1093/neuonc/noq178 21177781 PMC3064628

[18] Page MJ , McKenzie JE, Bossuyt PM, et al The PRISMA 2020 statement: an updated guideline for reporting systematic reviews. BMJ. 2021;372:n71. 10.1136/bmj.n71 33782057 PMC8005924

[19] Higgins J , Thomas J, Chandler J, et al Cochrane Handbook for Systematic Reviews of Interventions. Version 6.5. https://www.training.cochrane.org/handbook. Updated 2024. Accessed October 10, 2024.

[20] Critical Appraisal Skills Programme. CASP randomised controlled trial (RCT) checklist. CASP Web site. https://casp-uk.net/casp-tools-checklists/randomised-controlled-trial-rct-checklist/. Updated 2023. Accessed December, 2024.

[21] Critical Appraisal Skills Programme. CASP cohort study checklist. CASP Web site. https://casp-uk.net/casp-tools-checklists/cohort-study-checklist/. Updated 2023. Accessed December, 2024.

[22] Schünemann H , Brożek J, Guyatt G, Oxman A. GRADE handbook for grading quality of evidence and strength of recommendations. The GRADE Working Group Web site https://gdt.gradepro.org/app/handbook/handbook.html. Updated 2013. Accessed January, 2025.

[23] Leclair NK , Lambert W, Roche K, et al Early experience with targeted therapy as a first-line adjuvant treatment for pediatric low-grade glioma. Neuro­surg Focus. 2022;53:E15. 10.3171/2022.9.FOCUS22410 36455272

[24] Pérez JPM , Muchart J, López VS, et al Targeted therapy for pediatric low-grade glioma. Childs Nerv Syst. 2021;37:2511-2520. 10.1007/s00381-021-05138-3 33864514

[25] Kilburn LB , Khuong-Quang D, Hansford JR, et al The type II RAF inhibitor tovorafenib in relapsed/refractory pediatric low-grade glioma: the phase 2 FIREFLY-1 trial. Nat Med. 2024;30:207-217. 10.1038/s41591-023-02668-y 37978284 PMC10803270

[26] Mistry M , Zhukova N, Merico D, et al *BRAF* mutation and *CDKN2A* deletion define a clinically distinct subgroup of childhood secondary high-grade glioma. J Clin Oncol. 2015;33:1015-1022. 10.1200/JCO.2014.58.3922 25667294 PMC4356711

[27] Nobre L , Zapotocky M, Ramaswamy V, et al Outcomes of BRAF V600E pediatric gliomas treated with targeted BRAF inhibition. JCO Precis Oncol. 2020;4:10.1200/PO.19.00298. eCollection 2020 10.1200/PO.19.00298 PMC7446502 32923898

[28] Lassaletta A , Zapotocky M, Bouffet E. Chemotherapy in pediatric low-grade gliomas (PLGG). Childs Nerv Syst. 2024;40:3229-3239. 10.1007/s00381-024-06458-w 38819670

[29] Bouffet E , Geoerger B, Moertel C, et al Efficacy and safety of trametinib monotherapy or in combination with dabrafenib in pediatric BRAF V600-mutant low-grade glioma. JCO. 2023;41:664-674. 10.1200/JCO.22.01000 PMC9870224 36375115

[30] Andrews LJ , Thornton ZA, Saincher SS, et al Prevalence of BRAFV600 in glioma and use of BRAF inhibitors in patients with BRAFV600 mutation-positive glioma: systematic review. Neuro Oncol. 2022;24:528-540. 10.1093/neuonc/noab247 34718782 PMC8972326

[31] Cacciotti C , Tabori U, Hawkins C, Bennett J. Targeting the RAS/MAPK pathway in children with glioma. J Neurooncol. 2025;171:265-277. 10.1007/s11060-024-04857-2 39448518

[32] Garutti M , Bergnach M, Polesel J, Palmero L, Pizzichetta MA, Puglisi F. BRAF and MEK inhibitors and their toxicities: a meta-analysis. Cancers (Basel). 2022;15:141. 10.3390/cancers1501014110.3390/cancers15010141 36612138 PMC9818023

[33] Tsai JW , Choi JJ, Ouaalam H, et al Integrated response analysis of pediatric low-grade gliomas during and after targeted therapy treatment. Neurooncol Adv. 2023;5:vdac182. 10.1093/noajnl/vdac182 36926246 PMC10011805

[34] Selt F , Van Tilburg CM, Bison B et al Response to trametinib treatment in progressive pediatric low‑grade glioma patients. J Neurooncol. 2020;149: 499-510. 10.1007/s11060-020-03640-3 33026636 PMC7609413

[35] Manoharan N , Liu KX, Mueller S, Haas-Kogan DA, Bandopadhayay P. Pediatric low-grade glioma: targeted therapeutics and clinical trials in the molecular era. Neoplasia. 2023;36:100857. 10.1016/j.neo.2022.100857 36566593 PMC9803951

[36] Lassaletta A , Zapotocky M, Mistry M, et al Therapeutic and prognostic implications of BRAF V600E in pediatric low-grade gliomas. J Clin Oncol. 2017;35:2934-2941. 10.1200/JCO.2016.71.8726 28727518 PMC5791837

[37] O’Hare P , Cooney T, de Blank P, et al Resistance, rebound, and recurrence regrowth patterns in pediatric low-grade glioma treated by MAPK inhibition: a modified Delphi approach to build international consensus-based definitions—International Pediatric Low-Grade Glioma Coalition. Neuro Oncol. 2024;26:1357-1366. 10.1093/neuonc/noae074 38743009 PMC11300023

[38] Cipri S , Del Baldo G, Fabozzi F, Boccuto L, Carai A, Mastronuzzi A. Unlocking the power of precision medicine for pediatric low-grade gliomas: molecular characterization for targeted therapies with enhanced safety and efficacy. Front Oncol. 2023;13:1204829. 10.3389/fonc.2023.1204829 37397394 PMC10311254

[39] NICE. Dabrafenib with trametinib for treating BRAF V600E mutation-positive glioma in children and young people aged 1 year and over. National Institute for Health and Care Excellence (NICE) Web site. Updated 2024. https://www.nice.org.uk/guidance/ta977. Accessed January 2026. 40073183

[40] Pigott LE , Mileva K, Mancini L. Editorial: women in radiology: neuroimaging and neurotechnology. Front Radiol. 2025;5:1643898. https://www.frontiersin.org/journals/radiology/articles/10.3389/fradi.2025.1643898 40666633 10.3389/fradi.2025.1643898 PMC12260927

