# Supplementary material for: Outcomes of target therapies for pediatric BRAF V600-mutant low-grade gliomas: A systematic review and meta-analysis
Source: Neurooncol Adv. 2026 Jul 27;8(1):vdag192. doi: 10.1093/noajnl/vdag192 (PMC13499688; doi:10.1093/noajnl/vdag192)
Supplement: Supplementary file 1 [file vdag192_supplementary_data.docx]

**Supplementary Material**

**Supplementary Table 1-** PRISMA 2020 checklist

| **Section and Topic** | **Item #** | **Checklist item** | **Location where item is reported (Page nº)** |  |
| --- | --- | --- | --- | --- |
| **TITLE** | | |  | |
| Title | 1 | Identify the report as a systematic review. | 1 |  |
| **ABSTRACT** | | |  | |
| Abstract | 2 | See the PRISMA 2020 for Abstracts checklist. | 2-3 |  |
| **INTRODUCTION** | | |  | |
| Rationale | 3 | Describe the rationale for the review in the context of existing knowledge. | 4-6 |  |
| Objectives | 4 | Provide an explicit statement of the objective(s) or question(s) the review addresses. | 6 |  |
| **METHODS** | | |  | |
| Eligibility criteria | 5 | Specify the inclusion and exclusion criteria for the review and how studies were grouped for the syntheses. | 7 |  |
| Information sources | 6 | Specify all databases, registers, websites, organisations, reference lists and other sources searched or consulted to identify studies. Specify the date when each source was last searched or consulted. | 6-7 |  |
| Search strategy | 7 | Present the full search strategies for all databases, registers and websites, including any filters and limits used. | 6-7 |  |
| Selection process | 8 | Specify the methods used to decide whether a study met the inclusion criteria of the review, including how many reviewers screened each record and each report retrieved, whether they worked independently, and if applicable, details of automation tools used in the process. | 7 |  |
| Data collection process | 9 | Specify the methods used to collect data from reports, including how many reviewers collected data from each report, whether they worked independently, any processes for obtaining or confirming data from study investigators, and if applicable, details of automation tools used in the process. | 7 |  |
| Data items | 10a | List and define all outcomes for which data were sought. Specify whether all results that were compatible with each outcome domain in each study were sought (e.g. for all measures, time points, analyses), and if not, the methods used to decide which results to collect. | 7 |  |
|  | 10b | List and define all other variables for which data were sought (e.g. participant and intervention characteristics, funding sources). Describe any assumptions made about any missing or unclear information. | 7 |  |
| Study risk of bias assessment | 11 | Specify the methods used to assess risk of bias in the included studies, including details of the tool(s) used, how many reviewers assessed each study and whether they worked independently, and if applicable, details of automation tools used in the process. | 8 |  |
| Effect measures | 12 | Specify for each outcome the effect measure(s) (e.g. risk ratio, mean difference) used in the synthesis or presentation of results. | 8 |  |
| Synthesis methods | 13a | Describe the processes used to decide which studies were eligible for each synthesis (e.g. tabulating the study intervention characteristics and comparing against the planned groups for each synthesis (item #5)). | 7 |  |
|  | 13b | Describe any methods required to prepare the data for presentation or synthesis, such as handling of missing summary statistics, or data conversions. | 8 |  |
|  | 13c | Describe any methods used to tabulate or visually display results of individual studies and syntheses. | 8 |  |
|  | 13d | Describe any methods used to synthesize results and provide a rationale for the choice(s). If meta-analysis was performed, describe the model(s), method(s) to identify the presence and extent of statistical heterogeneity, and software package(s) used. | 8 |  |
|  | 13e | Describe any methods used to explore possible causes of heterogeneity among study results (e.g. subgroup analysis, meta-regression). | 8 |  |
|  | 13f | Describe any sensitivity analyses conducted to assess robustness of the synthesized results. | 8 |  |
| Reporting bias assessment | 14 | Describe any methods used to assess risk of bias due to missing results in a synthesis (arising from reporting biases). | 8 |  |
| Certainty assessment | 15 | Describe any methods used to assess certainty (or confidence) in the body of evidence for an outcome. | 8, 9-10 |  |
| **RESULTS** | | |  | |
| Study selection | 16a | Describe the results of the search and selection process, from the number of records identified in the search to the number of studies included in the review, ideally using a flow diagram. | 9 |  |
|  | 16b | Cite studies that might appear to meet the inclusion criteria, but which were excluded, and explain why they were excluded. | 9, Table 1 |  |
| Study characteristics | 17 | Cite each included study and present its characteristics. | 9 |  |
| Risk of bias in studies | 18 | Present assessments of risk of bias for each included study. | 9, Table 1 and S.Tab 4 |  |
| Results of individual studies | 19 | For all outcomes, present, for each study: (a) summary statistics for each group (where appropriate) and (b) an effect estimate and its precision (e.g. confidence/credible interval), ideally using structured tables or plots. | 11- 13  Fig.3,4,5  Table 2 |  |
| Results of syntheses | 20a | For each synthesis, briefly summarise the characteristics and risk of bias among contributing studies. | 11- 13 |  |
|  | 20b | Present results of all statistical syntheses conducted. If meta-analysis was done, present for each the summary estimate and its precision (e.g. confidence/credible interval) and measures of statistical heterogeneity. If comparing groups, describe the direction of the effect. | 11- 13 |  |
|  | 20c | Present results of all investigations of possible causes of heterogeneity among study results. | 13  S. Figs 1-4 |  |
|  | 20d | Present results of all sensitivity analyses conducted to assess the robustness of the synthesized results. | 13  S. Figs 4 |  |
| Reporting biases | 21 | Present assessments of risk of bias due to missing results (arising from reporting biases) for each synthesis assessed. | 13  S. Figs 4 |  |
| Certainty of evidence | 22 | Present assessments of certainty (or confidence) in the body of evidence for each outcome assessed. | 9, S.Tab 4 |  |
| **DISCUSSION** | | |  | |
| Discussion | 23a | Provide a general interpretation of the results in the context of other evidence. | 13-17 |  |
|  | 23b | Discuss any limitations of the evidence included in the review. | 17 |  |
|  | 23c | Discuss any limitations of the review processes used. | 17 |  |
|  | 23d | Discuss implications of the results for practice, policy, and future research. | 16-17 |  |
| **OTHER INFORMATION** | | |  | |
| Registration and protocol | 24a | Provide registration information for the review, including register name and registration number, or state that the review was not registered. |  |  |
|  | 24b | Indicate where the review protocol can be accessed, or state that a protocol was not prepared. |  |  |
|  | 24c | Describe and explain any amendments to information provided at registration or in the protocol. |  |  |
| Support | 25 | Describe sources of financial or non-financial support for the review, and the role of the funders or sponsors in the review. |  |  |
| Competing interests | 26 | Declare any competing interests of review authors. |  |  |
| Availability of data, code and other materials | 27 | Report which of the following are publicly available and where they can be found: template data collection forms; data extracted from included studies; data used for all analyses; analytic code; any other materials used in the review. |  |  |

**Supplementary Table 2**- PICO Framework

| **Population** | **Intervention/ Exposure** | **Comparison** | **Outcome Measure** |
| --- | --- | --- | --- |
| Paediatric patients (aged 1–17 years) with BRAF V600-mutant low-grade glioma (LGG). | Targeted therapies (BRAF inhibitors, MEK inhibitors, combination) | Alternative treatments (e.g. chemotherapy, radiotherapy) | 1. Progression-Free Survival (PFS) 2. Tumour response rate 3. Survival rate 4. Adverse effects 5. Tolerability 6. Short term functional outcomes |

**Supplementary Tables 3-** Summary of the search terms and Initial search results completed on the 20/12/24

**Table 3.1**- Summary of the search terms, combinations and Boolean operators included in the search strategy.

| **Key words** | **OR** | **Alternative words** |
| --- | --- | --- |
| 1) Paediatrics | OR | Pediatrics OR Children OR Adolescent OR Child* OR adolescen* OR young adult* |
| 2) "BRAF V600" | OR | "BRAF mutation" OR "Proto-Oncogene Proteins B-raf" [MeSH] OR "BRAF V600E" |
| 3) “Low-grade glioma” | OR | LGG |
| 4) "Targeted therapy" | OR | "BRAF inhibitors" OR "MEK inhibitors" OR "Molecular target therapy" [MeSH] OR "Protein Kinase Inhibitors" [MeSH] OR tovorafenib OR dabrafenib OR trametinib OR selumetinib OR Vemurafenib |
| 5) "Outcomes" | OR | Efficacy OR Safety OR "Quality of life" OR "Functional outcomes" OR "Treatment outcomes" |

**Table 3.2-** EBSCO- All databases

| NO. | Search Details | Filters applied | Results |
| --- | --- | --- | --- |
| #1 | (paediatric OR pediatric OR children OR adolescents OR Child* OR adolescen* OR young adult*) | --- | 18,969,594 |
| #2 | ("BRAF V600" OR "BRAF mutation" OR "Proto-Oncogene Proteins B-raf" OR "BRAF V600E") | --- | 21,895 |
| #3 | ("Low-grade glioma" OR LGG) | --- | 7,713 |
| #4 | ("targeted therapy" OR "BRAF inhibitors" OR "MEK inhibitors" OR "Molecular target therapy" OR "Protein Kinase Inhibitors" OR tovorafenib OR dabrafenib OR trametinib OR selumetinib OR Vemurafenib) | --- | 193,502 |
| #5 | (outcomes OR efficacy OR safety OR "quality of life" OR "Functional outcomes" OR "Treatment outcomes") | --- | 15,210,596 |
| 6# | #1 AND #2 AND #3 AND #4 AND #5 | --- | 25 |
| Total | | | 25 |

**Table 3.3-** Pubmed

| NO. | Search Detail | Filters applied | Results |
| --- | --- | --- | --- |
| #1 | Paediatric low-grade glioma AND Targeted therapy | From 2018 | 193 |
| #2 | (paediatric OR pediatric OR children OR adolescents OR Child* OR adolescen* OR young adult*) | --- | 560,388 |
| #3 | ("BRAF V600" OR "BRAF mutation" OR "Proto-Oncogene Proteins B-raf" OR "BRAF V600E") | --- | 1,669 |
| #4 | ("Low-grade glioma" OR LGG) | --- | 618 |
| #5 | ("targeted therapy" OR "BRAF inhibitors" OR "MEK inhibitors" OR "Molecular target therapy" OR "Protein Kinase Inhibitors" OR tovorafenib OR dabrafenib OR trametinib OR selumetinib OR Vemurafenib) | --- | 14,310 |
| #6 | (outcomes OR efficacy OR safety OR "quality of life" OR "Functional outcomes" OR "Treatment outcomes") | --- | 524,421 |
| #7 | #2 AND #3 AND #4 AND #5 AND #6 |  | 26 |
| Total | | | 219 |

**Table 3.4-** Science Direct

| NO. | Search Detail | Filters applied | Results |
| --- | --- | --- | --- |
| #1 | (paediatric OR pediatric OR children OR adolescents OR Child* OR adolescen* OR young adult*) AND ("BRAF V600" OR "BRAF mutation" OR "Proto-Oncogene Proteins B-raf" OR "BRAF V600E") AND ("Low-grade glioma" OR LGG) AND ("targeted therapy" OR "BRAF inhibitors" OR "MEK inhibitors" OR "Molecular target therapy" OR "Protein Kinase Inhibitors" OR tovorafenib OR dabrafenib OR trametinib OR selumetinib OR Vemurafenib OR ) AND ( outcomes OR efficacy OR safety OR "quality of life" OR "Functional outcomes" OR "Treatment outcomes") | --- | 0 |
| #2 | (pediatric OR children) | --- | 1,000,000+ |
| #3 | ("BRAF V600" OR "BRAF mutation") | --- | 14,356 |
| #4 | ("Low-grade glioma") | --- | 10,952 |
| #5 | ("targeted therapy") | --- | 141,799 |
| #6 | (outcomes) | --- | 1,000,000+ |
| #7 | #2 AND #3 AND #4 AND #5 AND #6 |  | 149 |
| Total | | | 149 |

**Table 3.5-** Cochrane

| NO. | Search Detail | Filters applied | Results |
| --- | --- | --- | --- |
| #1 | (paediatric OR pediatric OR children OR adolescents OR Child* OR adolescen* OR young adult*) AND ("BRAF V600" OR "BRAF mutation" OR "Proto-Oncogene Proteins B-raf" OR "BRAF V600E") AND ("Low-grade glioma" OR LGG) AND ("targeted therapy" OR "BRAF inhibitors" OR "MEK inhibitors" OR "Molecular target therapy" OR "Protein Kinase Inhibitors" OR tovorafenib OR dabrafenib OR trametinib OR selumetinib OR Vemurafenib OR ) AND ( outcomes OR efficacy OR safety OR "quality of life" OR "Functional outcomes" OR "Treatment outcomes") | --- | 0 |
| #2 | Paediatric AND "BRAF V600" AND "low-grade glioma" AND "targeted therapy" AND "functional outcomes" | --- | 0 |
| #3 | (pediatric OR children) AND ("BRAF V600" OR "BRAF mutation") AND ("low-grade glioma") AND ("targeted therapy") AND (outcomes) | --- | 0 |
| #4 | Paediatrics AND ("BRAF V600" OR "BRAF mutation") AND ("low-grade glioma" OR LGG) AND (dabrafenib OR trametinib OR selumetinib OR "targeted therapy") | --- | 0 |
| Total | | | 0 |

**Supplementary Table 4 -** GRADE Summary of findings for Efficacy and Safety of targeted therapies on pLGG.

| Outcome | Nº/ Study design | Risk of bias (CASP) | Inconsistency | Indirectness | Imprecision | Publication Bias | GRADE Certainty |
| --- | --- | --- | --- | --- | --- | --- | --- |
| ORR | 4 RCT | Low | not serious | not serious | not serious | None | ⨁⨁⨁⨁ High |
| ORR | 6 RCS | Moderate-High | Serious | not serious | serious | none | ⨁◯◯◯ Very low |
| PFS | 4 RCT | Low | not serious | not serious | not serious | none | ⨁⨁⨁⨁ High |
| PFS | 3/6 RCS | Moderate | Serious | not serious | not serious | none | ⨁◯◯◯ Very low |
| Grade 3/4 AEs | 4 RCT | Low | not serious | not serious | serious | none | ⨁⨁⨁◯ Moderate |
| Grade 3/4 AEs | 6 RCS | High | serious | not serious | serious | none | ⨁◯◯◯ Very low |

**Abbreviations:** RCT – Randomised Controlled Trial; RCS – Retrospective Cohort Study; NA – Not Available; MEKi – MEK Inhibitor; BRAFi – BRAF Inhibitor; PFS – Progression-Free Survival; ORR – Objective Response Rate; AE – Adverse Event;

**Supplement Figure 1 – Subgroup analysis for ORR**

**Figure 1A -** ORR- Subgroup analysis: Study design

**Figure 1B**- ORR- Subgroup Analysis: Therapy Type

**Supplementary Figure 2-** Subgroup Analysis for PFS

**Figure 2A-** PFS- Subgroup analysis: Study design

**Figure 2B-** PFS- Subgroup Analysis: Therapy Type

**Supplementary Figure 3-** Subgroup Analysis for Grade ¾ Adverse Events

**Figure 3A**- Grade 3/4 AEs- Subgroup analysis: Study design

**Figure 3B-** Grade 3/4 AEs- Subgroup analysis: Drug Class

**Figure 3C-** Grade 3/4 AEs- Subgroup analysis: therapy type

**Supplement Figure 4 -** Sensitivity Analysis

**Figure 4A-** Sensitivity Test: ORR- Exclusion of low-quality articles

**Figure 4B-** Sensitivity Test: ORR- Exclusion estimates ORR

**Figure 4C-** Sensitivity Test: PFS- Exclusion of low-quality articles

**Figure 4D -** Sensitivity Test: Grade 3/4 AEs- Exclusion of low-quality articles

**Figure Legends**

Figure 1- PRISMA 2020 flow diagram for new systematic reviews which included searches of databases, registers and other sources.

Table 1- Study characteristics and Quality Appraisal

Figure 2- Funnel plots and Egger’s test for publication bias

**(A)** Funnel plot for Overall Response Rate (ORR), showing treatment arms plotted by effect size (proportion) and standard error. Egger’s test (right) showed no significant small-study effects (p = 0.727).

**(B)** Funnel plot for Progression-Free Survival (PFS), with effect sizes expressed in months. Egger’s test showed marginal asymmetry (p = 0.071), suggesting a potential small-study effect.

**(C)** Funnel plot for Grade 3/4 Adverse Events (AEs), using proportion data. Egger’s test indicated no significant publication bias (p = 0.109).

Each blue dot represents a treatment arm. The red vertical line indicates the estimated pooled effect. Funnel symmetry supports the absence of publication bias, whereas asymmetry may indicate selective reporting.

Figure 3. Overall Response Rate (ORR) in paediatric BRAF V600-mutant LGG.

(A) Forest plot of ORR across 16 treatments arms using a random-effects model.

(B) Subgroup analysis of ORR by drug class (BRAF, Combination, MEK, other).

**Figure 4.** Progression-Free Survival (PFS) in paediatric BRAF V600-mutant LGG.

**(A)** Forest plot of pooled median PFS across 11 treatment arms, calculated using a random-effects model.

**(B)** Subgroup analysis of PFS by drug class (BRAF, Combination, MEK, other).

Figure 5 - Forest plot of Grade 3/4 Adverse Side Effects Across 10 treatments arms using a random-effects model

Table 2- Functional Outcomes Summary
